# Interobserver agreement on the sonographic severity grading of shoulder impingement syndrome

**DOI:** 10.1186/s13089-022-00272-8

**Published:** 2022-06-01

**Authors:** Raham Bacha, Syed Amir Gilani, Asif Hanif, Iqra Manzoor

**Affiliations:** 1 The University of Lahore, Lahore, Pakistan; 2 Gilani Ultrasound Center Affroasian Institute Lahore, Lahore, Pakistan

**Keywords:** Interobserver agreement, Shoulder impingement syndrome, Severity grading, Rotator cuff, Acromion-to-greater tuberosity distance

## Abstract

**Background:**

Shoulder impingement syndrome is the painful entrapment of the soft tissues between the acromion and the humeral head. The severity of shoulder impingement could be graded according to the limitation of shoulder joint moment. The reliability of sonographic findings in the grading of shoulder impingement severity grading is required to be evaluated by the consistency of findings between the observers.

**Purpose:**

To assess the interobserver agreement in the sonographic severity grading of shoulder impingement syndrome with the help of a ratio between acromion-to-greater tuberosity distance in the abduction and neutral arm position.

**Material and methods:**

Patients were examined by two independent observers in the coronal approach with neutral arm position. Acromion-to-greater tuberosity distance was measured in abduction and neutral shoulder position. The ratios of the distances in the abduction and neutral position were calculated to grade the severity of shoulder impingement syndrome.

**Results:**

A total of 78 shoulders were included in this study. A strong agreement was found for the grading of shoulder impingement severity grading between the two independent observers with Kappa value of 0.94. And correlation between the results of the two observers for the severity grading of shoulder impingement syndrome was significant at 0.01 level.

**Conclusion:**

Severity grading of the shoulder impingement syndrome was performed based on the ratio of acromion-to-greater tuberosity distance in abduction and neutral arm position. However, the sonographic findings were consistent and a strong interobserver agreement was seen in this sonographic severity grading.

**Supplementary Information:**

The online version contains supplementary material available at 10.1186/s13089-022-00272-8.

## Introduction

Shoulder impingement syndrome is one of the most common causes of shoulder pain, which is one of the most common musculoskeletal disorders [1]. Shoulder impingement or shoulder pain syndrome is the painful entrapment of the soft tissues between the acromion and the humeral head [2]. It could be caused either by narrowing of the shoulder outlet or thickening of its contents (supraspinatus tendon, subacromial subdeltoid bursa, joint capsule, etc.) [3]. The one-month prevalence of shoulder pain is between 16 and 30% [4]. Mainly there are two types of causes of shoulder pain syndrome, either there will be a reduction in the subacromial space or thickening of the contents of the shoulder outlet (supraspinatus tendon, glenohumeral ligaments, and subacromial subdeltoid bursa) [5]. For the evaluation of shoulder impingement syndrome, Neer and Hawkins tests are being used for a long. However plain X-ray, computed tomography, magnetic resonance imaging and ultrasound are being used for its evaluation as imaging modalities [6].

Dynamic sonography is progressively being used for the evaluation of shoulder impingement syndrome [7]. The moment of the supraspinatus and subacromial subdeltoid bursa could be observed in real-time while passing underneath the acromion during arm abduction [8]. No other imaging modality can evaluate structure in real-time during a physiologic moment [9, 10]. The sensitivity and specificity of ultrasound are high enough to be used as a gold standard for the assessment of shoulder impingement syndrome [7, 11]. Dynamic ultrasound proved as a helpful tool in the detection of various abnormalities of the painful shoulder especially impingement syndrome [12]. In cases of full-thickness tear, the sensitivity and specificity of ultrasound are very high 100% and 97%, respectively, whereas slightly low in partial-thickness tears [13]. The reproducibility of the radiation-based modalities is better, however, having a high potential for bioeffects [14]. In contrast to other imaging modalities, ultrasound is a non-invasive, inexpensive, readily available, relatively quick procedure, with no special preparation required, and safe for the diagnosis of musculoskeletal disorders [15, 16]. However, sonoelastography has been increasingly used to investigate musculoskeletal disorders [17].

The severity of the shoulder impingement syndrome is linked with the range of shoulder moment restrictions. Therefore, the severity of the shoulder impingement syndrome is classified in different severity grades ranging from Grade-0 to Grade-3. Neer classification of shoulder impingement was done as: acute inflammation, edema, hemorrhage in the rotator cuff in patients younger than 25 years was termed as Grade-1, fibrosis, and tendinitis of the rotator cuff usually between 25 and 40 years was termed as Grade-2, while mechanical disruption of the rotator cuff tendons, changes in the coracoacromial arch and osteophytes along the acromion in patients more than 40 years was Grade-3 [4]. The management plane of the shoulder impingement syndrome is generally based on the degree of functional disturbance, and the extent of structural damage [18, 19]. In the current study, the interobserver agreement is checked on the degree of functional disability categorized into four grades.

## Materials and methods

**Fig. 1:**
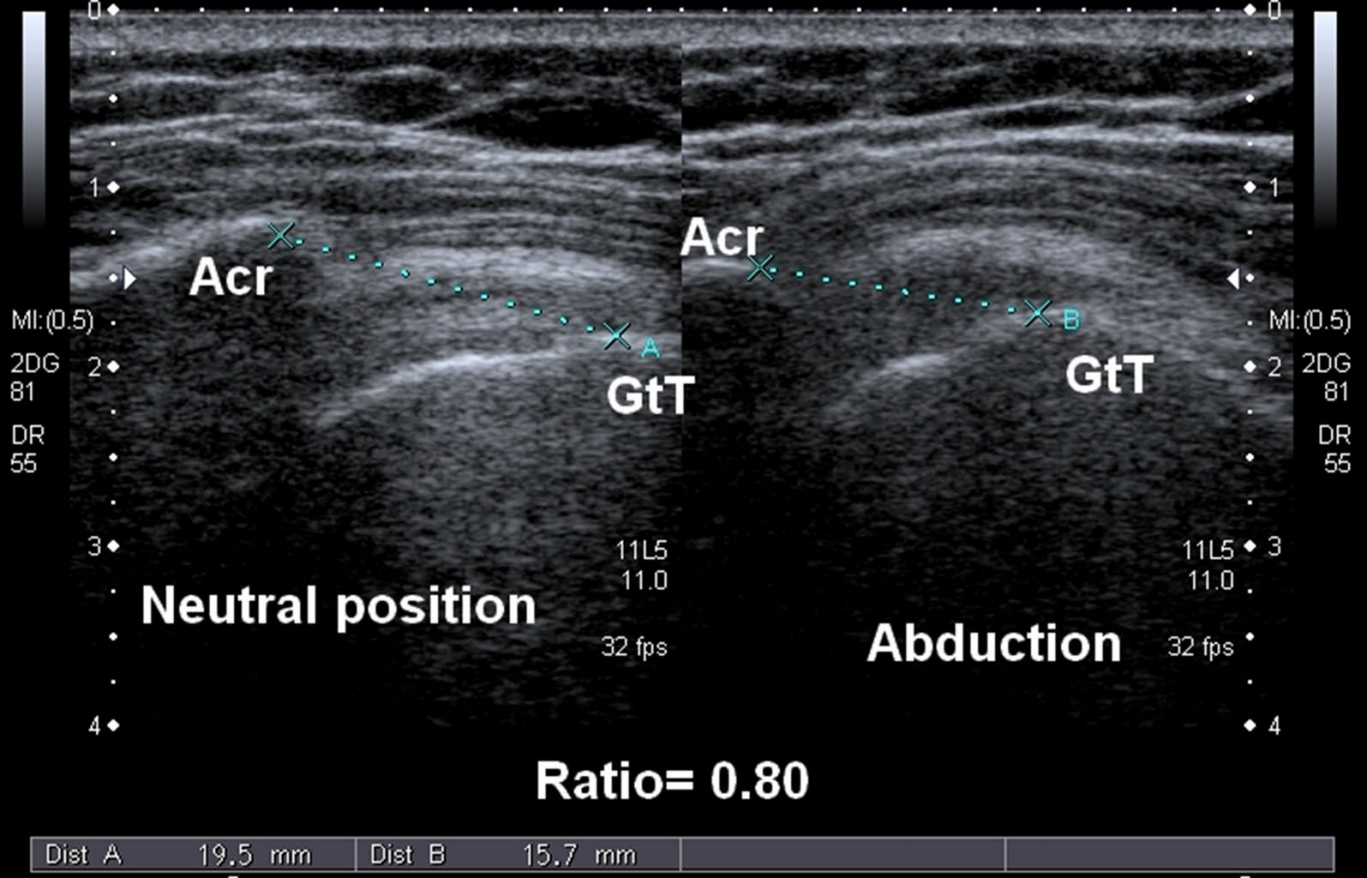
Left shoulder of a 37-year-old female with Grade-3 impingement scanned by first observer. Acromion-to-greater tuberosity distance in neutral position was 19.5 mm while in abduction it was 15.7 mm. The ratio between abduction and neutral position of the acromion-to-greater tuberosity distance was 0.80

**Fig. 2:**
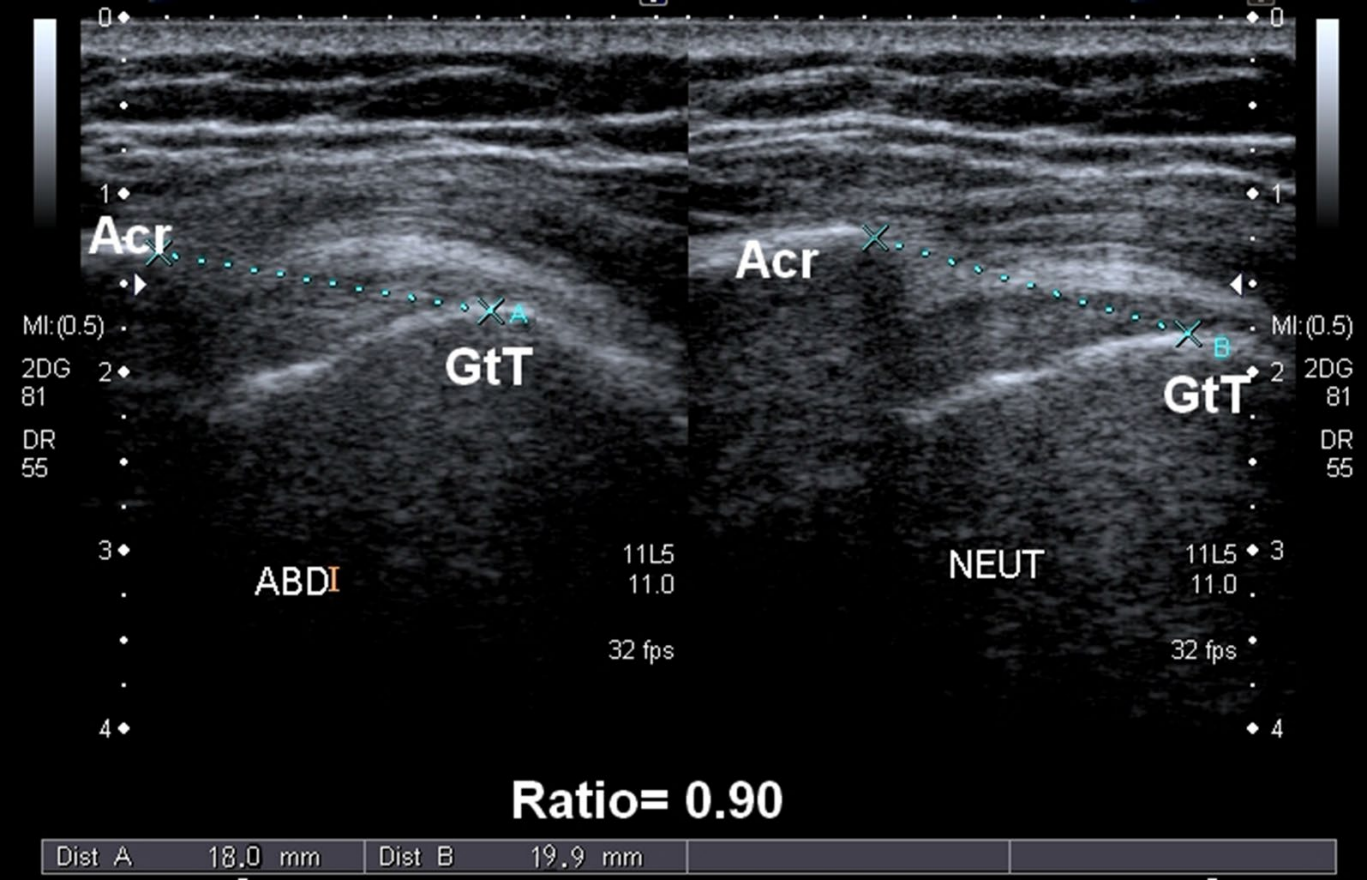
Left shoulder of the same female patient shown in Fig. 1, with Grade-3 impingement scanned by second observer. Acromion-to-greater tuberosity distance in neutral position was 19.9 mm while in abduction it was 18.0 mm. The ratio between abduction and neutral position of the acromion to greater tuberosity distance was 0.90. *Acr* Acromion, *GtT* Greater tuberosity, *ABD* Abduction, *NEUT* Neutral Position

It was a cross-sectional observational study conducted in 2020 at Gilani Ultrasound Center, Lahore, Pakistan. A total of 78 shoulders were included, comprising 56 (71.80%) with positive dynamic sonography while 22 (28.20%) were negative. All the patients with shoulder surgeries were excluded. The study was aimed to check for the interobserver agreement for the severity grades of the shoulder impingement syndrome with the help of high-resolution ultrasonography. Approval was taken from the institutional review board (IRB) and the Ethical Committee of the University of Lahore. A single ultrasound unit Toshiba Xario with linear transducer frequency ranging from 7 to 14 MHz was used for this study. Patients have been explained the procedure and aim of the research and written informed consent was signed. American Institute of Ultrasound in Medicine (AIUM) guidelines for shoulder ultrasound scanning were followed in this study [20]. Acromion-to-greater tuberosity distance was measured in the neutral position from the most prominent, from the palpable lateral margin of the acromion to the prominent superior facet of the greater tuberosity, while the elbow flexed at 90 degrees and hand is placed on the same thigh. The linear transducer was placed in coronal view, while its one end is placed on the lateral margin of the acromion and the other on the superior facet of the greater tuberosity both of them appear echogenic on ultrasound. Then the patient was asked for abducting the arm in internal rotation while the elbow is flexed at 90 degrees and the transducer is placed in the same position. The level of abduction at which the relative moment of the acromion and greater tuberosity was ceased and the patient feels pain in the shoulder, the image was frozen and the measurement was taken again between the same bony prominences (Figs. 1, 2). The severity of the shoulder impingement syndrome was graded based on the ratio of the difference between the distances at arm abduction and neutral position. The same procedure was repeated on the same patient one by one, while each observer examined the patient in the absence of the other. However, shoulder impingement syndrome was graded in three grades of severity based on the ratio of the acromion-to-greater tuberosity distance during abduction and neutral position. The individuals whose greater tuberosity disappears underneath the acromion during abduction have no measurable distance and their ratio becomes zero. Those individuals were normal, having no evidence of shoulder impingement syndrome. However, the ratio 0.01 (1%) to 0.4 (40%) was termed as minor impingement of Grade-1. The ratio is 0.4 (40%) to 0.7 (70%) was labeled as Grade-2 impingement, while the ratio greater than 0.70 (70%) was termed as Grade-3 impingement. These calculations were done on the acquired data by two independent, experienced sonologists (Figs. 1, 2). Statistical Package for the Social Sciences (SPSS) version 24 (SPSS 24, IBM, Armonk, NY, United States of America) software was used for the evaluation of data [21]. The interobserver agreement was measured with the help of Kappa test. The frequency of the shoulder impingement based on the dynamic sonography test was calculated. The mean of the acromion to greater tuberosity in abduction and neutral position and its ratio obtained by both the observers were compared.

## Results

A total of 78 shoulders were included in this research while 43 (55.12%) shoulders were of male and 35 (44.87%) female participants. The mean age of all the patients was 40.67 ± 12.75 years (10–74). The mean age of all the participants having no shoulder impingement was 38.14 ± 12.00 years (21–72), while the mean age of all the patients having mild shoulder impingement of Grade-1 severity was 44.33 ± 13.70 years (27–74). But the mean age of all the patients having moderate shoulder impingement of Grade-2 severity was 42.74 ± 10.10 years (24–60). However, the mean age of all the patients having severe shoulder impingement of Grade-3 severity was 36.88 ± 14.53 years (10–58). Among all the shoulders, 43 (53.1%) were right while 35 (43.2%) were left. Besides, 22 (27.2%) were normal, while 21 (25.9%) were suffering from severity Grade-1, 19 (23.5%) from Grade-2, and 16 (19.8%) from Grade-3 shoulder impingement.

Acromion-togreater tuberosity distance during abduction and neutral position was measured by both the observers, but a slight difference was seen in their measurements. However, very small or even no difference was observed between the acromion-to-greater tuberosity distance in abduction and neutral position ratio (Table 1, Figs. 1, 2). The severity grading of shoulder impingement is based on this ratio; however, a strong agreement was found for the grading of between the two independent observers with Kappa value of 0.94. And the correlation between the results of the two observers for the severity grading of shoulder impingement syndrome was significant at 0.01 level. Details of the agreement of observer-1 and observer-2 for the grading of shoulder impingement severity in three distinct grades are given in Table 2.

**Table 1:** Comparison of the means of acromion-to-greater tuberosity distance in neutral position and abduction of the arm and its ratio in various grades of shoulder impingement syndrome, observed by observer-1 and observer-2

Severity grades	Observer 1			Observer 2		
	AGT D Nut	AGT D Abd	Ratio	AGT D Nut	AGT D Ab	Ratio
Grade 0						
*N*	22	22	22	22	22	22
Mean	14.74	0.00	0.00	16.76	0.00	0.00
Std. deviation	1.00	0.00	0.00	1.12	0.00	0.00
Minimum	13.66	0.00	0.00	14.70	0.00	0.00
Maximum	17.60	0.00	0.00	19.60	0.00	0.00
Grade 1						
*N*	20	20	20	20	20	20
Mean	17.25	5.71	0.33	17.27	5.75	0.33
Std. deviation	3.03	1.51	0.06	2.68	1.52	0.06
Minimum	13.46	3.90	0.19	14.00	3.67	0.20
Maximum	22.55	9.30	0.41	23.10	9.31	0.40
Grade 2						
*N*	20	20	20	20	20	20
Mean	15.92	8.36	0.52	15.92	8.32	0.53
Std. deviation	1.96	1.41	0.09	2.02	1.45	0.09
Minimum	13.68	6.14	0.40	12.00	5.38	0.40
Maximum	22.20	11.39	0.71	21.25	10.85	0.70
Grade 3						
*N*	16	16	16	16	16	16
Mean	14.81	12.17	0.82	15.90	12.81	0.81
Std. deviation	2.25	1.91	0.08	1.85	1.83	0.07
Minimum	13.33	10.08	0.72	12.40	9.50	0.71
Maximum	22.53	16.33	0.99	21.32	15.45	0.95
Total						
*N*	78	78	78	78	78	78
Mean	15.70	6.11	0.39	16.50	6.24	0.39
Std. deviation	2.35	4.63	0.30	2.03	4.80	0.30
Minimum	13.33	0.00	0.00	12.00	0.00	0.00
Maximum	22.55	16.33	0.99	23.10	15.45	0.95

AGT D = acromion-to-greater tuberosity distance, Nut = neutral position, Abd = abduction of the arm, *N* = number of individuals, Std. = standard

**Table 2:** Cross-tabulation to find the agreement of observer-1 and observer-2 in the shoulder impingement severity grading

Observer 1	Observer 2				Total
	Grade 0	Grade 1	Grade 2	Grade 3	
Grade 0					
Count	22	0	0	0	22
Expected count	6.2	5.6	5.6	4.5	22
Grade 1					
Count	0	19	1	0	20
Expected count	5.6	5.1	5.1	4.1	20
Grade 2					
Count	0	1	18	0	19
Expected count	5.4	4.9	4.9	3.9	19
Grade 3					
Count	0	0	1	16	17
Expected count	4.8	4.4	4.4	3.5	17
Total					
Count	22	20	20	16	78
Expected count	22	20	20	16	78

## Discussion

Pain, weakness, and loss of movement at the shoulder due to irritation of the soft tissues as they pass underneath the acromion are due to the inflammation of tendons, bursa, and joint capsule [5, 22]. Plain X-ray is unable to diagnose soft tissue-related abnormalities; however, ultrasound is the modality of choice to observe cortical parts of the bone as well as soft tissue-related abnormalities [23]. The addition of dynamic ultrasound examination for the diagnosis of the painful shoulder showed the highest sensitivity in the assessment of impingement syndrome. However, 85.7% sensitivity for rotator cuff partial-thickness tear and 90% for rotator cuff full-thickness tear [12]. Shoulder impingement or shoulder pain syndrome is more clinical and it is not the expertise of ultrasound to merely declare the presence or absence of impingement syndrome. Rather it is important to explore the underlying cause of this syndrome for proper management and treatment plan [24].

The severity grading of the shoulder impingement syndrome is crucial for the selection of the management plane. While abducting the arm from the neutral position (vertical) to the level of the shoulder (horizontal) there are almost a rotation of 90 degrees. In the current study, this rotation is divided into three sets with arm abduction. The total distance from the acromion to greater tuberosity in the neutral position is taken equivalent to 90-degree rotation. If the entrapment occurs in the terminal 40% of the rotation, it was labeled as Grade-1 or minor impingement. But the entrapment of structures from 40 to 70% of the rotation was declared as Grade-2 or moderate impingement. However, the locking of shoulder moment at more than 70% of the total rotation was suggested as Grade-3 impingement (Additional file 1: Video). According to a study, Grade-1 shoulder impingement was assigned to the clinical conditions of tendinitis, tendon degeneration and partial tear with a simple painful shoulder, while calcific tendinitis, bursitis and adhesive capsulitis were included in Grade-2 shoulder impingement. However total tears of the rotator cuff and biceps tendon were termed as Grade-3 shoulder impingement [25]. Based on shoulder movement and sonographic appearance, impingement was categorized into the following grades in a study. If there is neither pain while moving the shoulder nor sonographic evidence of impingement, then it was graded as Grade-0. Pain during shoulder moment while sonographically there was no evidence of impingement then it was graded as 1. Pain during shoulder moment and sonographic evidence of impingement then it was graded as 2. However, Pain during shoulder moment while sonographic evidence of upward migration of the humeral head was graded as 3. Dynamic sonography was declared an imaging test of choice for the diagnosis of shoulder impingement syndrome [26].

The main objective of the current study was to assess the interobserver agreement on the severity grading of the shoulder impingement syndrome. The severity grading was purely made on the basis of the ratio of the acromion-to-greater tuberosity distance in abduction and neutral position. However, it was observed that there was a substantial difference in the means of acromion-to-greater tuberosity distance measured by both the sonologists. Whatever the cause may be, most probably due to slight variation in transducer placement and maneuvering, a strong agreement was observed between the independent sonologists in the measurement of the ratio between acromion to grater tuberosity distance during abduction and neutral position. Ultimately, there was a strong agreement between the sonologists on the grading. The Kappa value for the agreement was 0.94, which lied at the level of a strong agreement. In other words, there was a strong correlation between the findings of both the sonologists, related to the shoulder impingement severity grading, the Pearson correlation was significant at 0.01 (Figs. 1, 2). A study was conducted to assess the interobserver agreement in the examination of rotator cuff tendons through sonography. Kappa value of more than 0.60 shows a good agreement among the observers for the diagnosis of a full-thickness rotator cuff tear, while the p-value was significant at 0.01 level, means there was a strong correlation among the findings of the observers. However, the agreement among the experienced and inexperienced examiners was not strong. Therefore, the existing criteria of shoulder pain syndrome were operator-dependent. Therefore, a more clearly defined training curriculum and training programs were recommended by the author [27].

## Conclusion

Severity grading of the shoulder impingement syndrome was performed based on the ratio of acromion-to-greater tuberosity distance in abduction and neutral arm position. However, the sonographic findings were consistent and a strong interobserver agreement was seen in this sonographic severity grading.

## Supplementary Information

**Additional file 1: Video.** Dynamic evaluation of normal shoulder during abduction and various grades of impingement. In normal shoulders, greater tuberosity and rotator cuff tendon along with subacromial subdeltoid bursa were smoothly moved underneath the acromion during arm abduction. In Grade-1 impingement, Supraspinatus tendon along with bursa and greater tuberosity were entrapped to move, however there was a minimal restriction of the shoulder moment. In Grade-2 impingement, Supraspinatus tendon along with bursa and greater tuberosity were entrapped to move with a mid-range of moment, however there was a moderate restriction of the shoulder moment. In Grade-3 impingement, Supraspinatus tendon along with bursa and greater tuberosity were entrapped to move with a slight detectable moment, however there was a severe restriction of the shoulder moment.

## Data Availability

Data are available on demand.
